# Modulation of Macrophage Polarization by Carbon Nanodots and Elucidation of Carbon Nanodot Uptake Routes in Macrophages

**DOI:** 10.3390/nano11051116

**Published:** 2021-04-26

**Authors:** Andrew Dunphy, Kamal Patel, Sarah Belperain, Aubrey Pennington, Norman H. L. Chiu, Ziyu Yin, Xuewei Zhu, Brandon Priebe, Shaomin Tian, Jianjun Wei, Xianwen Yi, Zhenquan Jia

**Affiliations:** 1 Department of Biology, The University of North Carolina at Greensboro 312 Eberhart Building, 321 McIver Street, Greensboro, NC 27402-617, USA; amdunphy@uncg.edu (A.D.); kkpatel3@uncg.edu (K.P.); srbelper@uncg.edu (S.B.); a_pennin@uncg.edu (A.P.); bmpriebe@uncg.edu (B.P.); 2 Department of Chemistry and Biochemistry, University of North Carolina at Greensboro, Greensboro, NC 27412, USA; nhchiu@uncg.edu; 3 Department of Nanoscience, Joint School of Nanoscience and Nanoengineering, University of North Carolina at Greensboro, Greensboro, NC 27401, USA; z_yin@uncg.edu (Z.Y.); j_wei@uncg.edu (J.W.); 4 Department of Internal Medicine, Section on Molecular Medicine, Wake Forest School of Medicine, Winston-Salem, NC 27101, USA; xwzhu@wakehealth.edu; 5 Department of Microbiology & Immunology, University of North Carolina, Chapel Hill, NC 27599, USA; shaomin_tian@med.unc.edu; 6 Lineberger Comprehensive Cancer Center, University of North Carolina, Chapel Hill, NC 27599, USA; xianwen_yi@med.unc.edu; 7 McAllister Heart Institute, University of North Carolina, Chapel Hill, NC 27599, USA

**Keywords:** carbon nanodots, macrophages, polarization, phagocytosis, uptake routes

## Abstract

Atherosclerosis represents an ever-present global concern, as it is a leading cause of cardiovascular disease and an immense public welfare issue. Macrophages play a key role in the onset of the disease state and are popular targets in vascular research and therapeutic treatment. Carbon nanodots (CNDs) represent a type of carbon-based nanomaterial and have garnered attention in recent years for potential in biomedical applications. This investigation serves as a foremost attempt at characterizing the interplay between macrophages and CNDs. We have employed THP-1 monocyte-derived macrophages as our target cell line representing primary macrophages in the human body. Our results showcase that CNDs are non-toxic at a variety of doses. THP-1 monocytes were differentiated into macrophages by treatment with 12-*O*-tetradecanoylphorbol-13-acetate (TPA) and co-treatment with 0.1 mg/mL CNDs. This co-treatment significantly increased the expression of CD 206 and CD 68 (key receptors involved in phagocytosis) and increased the expression of CCL2 (a monocyte chemoattractant and pro-inflammatory cytokine). The phagocytic activity of THP-1 monocyte-derived macrophages co-treated with 0.1 mg/mL CNDs also showed a significant increase. Furthermore, this study also examined potential entrance routes of CNDs into macrophages. We have demonstrated an inhibition in the uptake of CNDs in macrophages treated with nocodazole (microtubule disruptor), N-phenylanthranilic acid (chloride channel blocker), and mercury chloride (aquaporin channel inhibitor). Collectively, this research provides evidence that CNDs cause functional changes in macrophages and indicates a variety of potential entrance routes.

## 1. Introduction

Cardiovascular disease (CVD) has more clinical implications than any other condition worldwide. Globally, CVD accounts for one-third of all deaths [1]. In the United States, over 600,000 humans die of CVD per year, representing a quarter of all American deaths [2]. For these reasons, devoting resources and research into ameliorating the mortality caused by CVD is of principal priority. CVD can be expressed in several types such as ischemic heart disease, cerebrovascular disease, or coronary artery diseases, which is usually caused by atherosclerosis [3]. Atherosclerosis is the build-up of plaque in artery walls. This condition leads to a decrease in blood flow to tissues. With the ever-increasing mortality rate due to CVD, it is crucial to develop new methods of treatment. The biggest challenge still is understanding the ramifications of the development of atherosclerosis.

Macrophages play a key role in the onset of the disease state. Free oxygen radicals modify low-densitiy lipoprotieins (LDL) into ox-LDL. Upon injury to the endothelium of blood vessels by ox-LDL, circulating monocytes differentiate into pro-inflammatory (M1) or anti-inflammatory (M2) macrophages. In progressing lesions, M1 macrophages engulf excess ox-LDL. In the process, these macrophages become lipid-laden and lose mobility, finally proceeding to settle en masse on the bed of arteries as plaque. Dysregulated plaque build-up in arteries results in a several fatal long-term health issues. Due to their crucial role as mediators in atherogenesis, as well as their involvement in several aspects of the immune response, macrophages are popular targets in vascular research and therapeutic treatment. 

In recent years, interest in the development of nanoparticles for biological application has risen. A key area of intrigue revolves around the interaction of nanoparticles with some aspects of the immune response, resulting in their induction or repression [4]. Their use also extends to imaging macrophages and disease states such as atherosclerotic lesions [5,6]. Carbon nanodots (CNDs) are particles of particular interest for a variety of reasons. These particles tend to be smaller than 10 nm in size, have an sp2 hybridization, and are quasi-spherical [7]. An essential characteristic of these nanodots is their high hydrophilicity, which is made apparent by the presence of several functional groups in their surface such as ether, carbonyl, hydroxyl, etc. This hydrophilicity allows for a very biocompatible particle that is ready to interact with various organic or inorganic species [7]. CNDs also have photoluminescent properties. This, combined with their hydrophilicity, makes CNDs useful in sensing other particles. Their luminescent characteristics are defined by their individual size, shape, functional groups, and other factors. Upon excitation by UV to visible light, CNDs emission wavelengths range from UV to near-infrared [7].

CNDs have been synthesized with scavenging properties and proved themselves capable ex vivo scavengers of free radicals, one of which is 2,2-diphenyl-1-picrylhydrazyl radicals (DPPH) [8]. In this assay, the successful conversion of DPPH into a stable DPPH-H complex is due to antioxidant activity. This leads to a change in color from violet to light yellow, which can be quantified by ultraviolet-visible spectroscopy. Zhang et al. demonstrated a dose-dependent increase in DPPH scavenging using N,S-codoped CNDs [8]. These nanoparticles were synthesized using citric acid, α-lipoic acid, and urea precursors through a hydrothermal method [9]. In vitro, CNDs have also demonstrated radical scavenging ability. By way of Di-Chloro Di-Hydrofuran Fluorescein Di-Acetate (DCFH-DA) assay and NBT (Nitro Blue Tetrazolium) reduction assay, Das et al. showcased CNDs (synthesized by microwave irradiation of date molasses) scavenging ability of hydroxyl and superoxide free radicals [10]. Altogether, these results denote the antioxidant propensity of CNDs and evidence their potential for biological utilization.

Atherosclerosis is a long-standing inflammatory disease characterized by the narrowing of arteries due to a build-up of plaque. The overproduction of ROS and its subsequent oxidative stress play a key role in its initiation. Macrophages play an essential role as intermediators of the disease state by differentiating into a pro-inflammatory state, secreting cytokines and eventually becoming foam cells. As the concrete source for plaque build-up, macrophages signify an area of interest. CNDs are a prospective choice for biomedical implementation, having shown usefulness in ROS scavenging, biosensing, and drug delivery. However, currently, no account exists that indicates whether or not CNDs have any ability to affect the M1/M2 polarization of macrophages. In this study, we examined the effects of CNDs on the expression of M1/M2 biomarkers and phagocytic activity of macrophages, as well as potential entrance routes.

## 2. Materials and Methods

### 2.1. Cell Culture

The THP-1 cell line (ATCC^®^ TIB-202^™^, Manassas, VA, USA) cells were cultured in Roswell Park Memorial Institute 1640 Medium (RPMI 1640) fortified with 10% Fetal Bovine Serum (FBS) and 1% Penicillin–Streptomycin. This cell line was obtained from the ATCC (Manassas, VA, USA) and grown in Cellstar^®^ Filter Cap 75 cm^2^ cell-culture treated, filter screw cap flasks in humidified incubators programmed to 37 °C and 5% CO_2_. Corresponding media was renewed every 2 days and cells were split into a new passage upon 85–90% confluence.

### 2.2. CNDs Synthesis and Characterization

The CNDs preparation using citric acid and ethylenediamine (EDA) as precursors was synthesized based on an adaption of a previously published microwave-assisted method [11]. In a 100 mL beaker, 0.96 citric acid 99.5% (Sigma-Aldrich, St. Louis, MO, USA) was dissolved with 10.0 mL DDI water and then mixed with 1 mL (0.8980 g/mL) EDA 99% (ACROS Organics, Fair Lawn, NJ, USA) under vigorous stirring for 30 s. The beaker containing the clear and colorless solution was covered with a watch glass and heated using a domestic microwave oven (1200 W) for 5.0 min. After the elapsed time, the beaker and contents were allowed to cool to room temperature. The brownish-orange crystalline product was diluted with 10.0 mL DDI water and then dialyzed using a 500–1000 Da MWCO Spectra Por Float-A-Lyzer G2 (Repligen, Waltham, MA, USA). The resultant clear, brownish-orange aqueous solution was lyophilized using Labconco FreeZone Plus 12 (Labconco, Kansas City, MO, USA) to obtain the dried carbon nanodot product. UV-Vis spectroscopy of CNDs was performed by Cary^®^ Eclipse TM Fluorescence Spectrophotometer (Agilent, Santa Clara, CA, USA). Upon dilution to 2 mg/mL in DI-H_2_O, CNDs were measured for fluorescence in a quartz cuvette to determine excitation and emission wavelengths. The surface chemistry of CNDS was characterized by carbon 1s X-ray photoelectron spectroscopy (XPS, ESCALAB 250 Xi, Thermo Fisher, West Sussex, UK). 

### 2.3. Monocyte Differentiation into Macrophages

THP-1 cells were cultured in cell plates containing RPMI 1640 Medium (Sigma-Aldrich, St. Louis, MO, USA) or Hank’s Balanced Salt Solution (HBSS) with calcium, magnesium, and glucose (Sigma-Aldrich, St. Louis, MO, USA). Monocyte differentiation into macrophages was induced with 3 ng/µL of 12-O-tetra-decanoylphorbol-13-acetate (TPA) in an incubation period of 72 h period. Cells were lifted by cell scraper and the supernatant placed in 50 mL Falcon tubes. Cell plates were rinsed with 2 mL PBS and also added to the supernatant. Then, cells were pelleted by centrifuge.

### 2.4. CNDs Treatment

For cell viability measured by trypan blue staining and flow cytometry ViaCount assays, THP-1 cells were treated with 3 ng/mL TPA in the presence or absence of 0.01, 0.1, 0.3, or 0.6 mg/mL CNDs in RPMI media for 72 h. The media was refreshed, and then, the cells were incubated for another 72 h. For other experiments, THP-1 monocytes were cultured in cell plates and co-treated with 0.1 mg/mL CNDs and 3 ng/µL TPA for 72 h in RPMI media. Incubation occurred in 37 °C/5% CO_2_ incubators. Surrounding media was decanted and replaced with new media, followed by another incubation period of 72 h, after which plates were rinsed with 2 mL PBS and also added to the supernatant. Then, cells were pelleted by centrifuge.

### 2.5. Cell Count (Trypan Blue)

Before and after differentiation and CNDs treatments, cells were counted. Monocytes and macrophages were centrifuged at 300 *g* for 5 min at 4 °C and resuspended in either PBS or respective media. After resuspension, cells were counted using a hemocytometer. Trypan blue was used to count viable, unstained cells, and the resulting concentration was also calculated.

### 2.6. RNA Extraction

THP-1 cells were cultured in appropriate media in Corning^®^ cell culture treated plates (Corning Life Sciences, Durham, NC, USA). Upon treatment and incubation, adhered cells were lifted sing a cell scraper. The media was extracted into 50 mL tubes. The cell plates were rinsed twice with 1 × PBS to ensure no treatment media remained and also to extract any remaining cells. Then, cells were then centrifuged at 300 *g* for 5 min at 4 °C. The supernatant media was decanted, and the resulting cell pellets were treated with 1 mL of ambion TRIzol^®^ (Thermo Fisher Scientific, Waltham, MA, USA). The resulting solution was pipetted into 1 mL Eppendorf tubes. Then, 200 µL of chloroform were added, which was followed by agitation, and then the solution was centrifuged at 12,000 rcf for 15 min. The top aqueous phase was transferred to another set of 1 mL Eppendorf tubes and then combined with 500 µL isopropanol and agitated before centrifuging again at 12,000 rcf for 10 min. The resulting pellet (RNA) was washed with 1 mL 75% ethanol and centrifuged at 7400 rcf for 5 min twice. Then, the pellet was resuspended in 10–15 µL of DEPC H_2_O.

### 2.7. CDNA Synthesis

After RNA extraction, the resulting RNA was quantified by way of a Thermo Scientific™ Nanodrop 2000 (Thermo Fisher Scientific, Waltham, MA, USA). Then, RNA was diluted to a concentration of 500 ng/µL. Then, 2 µL of diluted RNA were mixed with 5 µL of 5× Buffer, 1.25 µL of ddNTP, 1.25 µL of Random Primer, 14.875 µL of DEPC H_2_O, and 0.625 µL of MMLV-Reverse Transcriptase. Using Applied Biosystems™ Veriti™ 96-Well Thermal Cycler (Thermo Fisher Scientific, Waltham, MA, USA), the 25 µL solution was converted to cDNA.

### 2.8. Quantitative Real-Time Polymerase Chain Reaction

Once cDNA was synthesized using the methods above, the resulting cDNA was probed for a selection of M1/M2 biomarkers as mentioned previously, using H_GAPDH as a housekeeping gene. This was performed by mixing 1 µL of cDNA with 10 µL of Power SYBR^®^ Green PCR Master Mix, 2 µL of 5 µM Forward Primer (Table 1), 2 µL of 5 µM Reverse Primer (Table 1), 2 µL of 1:10 diluted cDNA, and 5 µL of DEPC H_2_O. The Applied Biosystems™ StepOnePlus™ Real-Time PCR system (Thermo Fisher Scientific, Waltham, MA, USA) was employed and ran for 40 cycles. Each individual cycle constituted a 95 °C phase for 15 s, a 58 °C phase for 60 s, and a 60 °C phase for 15 s. Comparative threshold values were evaluated in order to quantify gene expression.

### 2.9. Vybrant™ Phagocytosis Assay Kit (V-6694)

First, 4 × 10^6^ THP-1 cells were grown in cell culture plates in corresponding media. Differentiation into macrophages was induced by administering 3 ng/µL TPA with an incubation period of 72 h (with or without co-treatment with a CNDs concentration of 0.1 mg/mL) at 37 °C, 5% CO2. After harvesting and pelleting cells, concentration was re-suspended in HBSS to 2 × 10^6^ cells/mL. Then, 1 mL of control cells were treated with 1000 ng/mL TPA in order to activate cells to serve as a positive control. Next, 100 µL of cell suspension was added to 5 wells per sample, plus 50 µL of HBSS (negative control wells contained 200 µL of HBSS). Cells were left to incubate for 18 h in 35 °C/5% CO_2_ incubators. This incubation period allows macrophages to settle. Then, HBSS was removed, and 200 µL of fluorescently labeled *E. coli* suspension was administered for 2 h. Upon the removal of suspension, cells were treated for 60 s with 100 µL of Trypan Blue suspension. Immediate removal of suspension followed. The phagocytic activities of cells were quantified using a BioTek™ Synergy 2.0 plater reader (BioTek Instruments, Winooski, VT, USA).

### 2.10. ViaCount Flow Cytometry

Cells were cultured with the necessary incubation times and treatments. Next, cells were harvested by cell scraping and placed into tubes. The cell concentration was adjusted to 5 × 10^6^/mL. Then, 80 µL of cells were treated with 20 µL of ViaCount Reagent for 10 min at room temperature, upon which 500 µL of cold PBS was added. The samples were analyzed for viability using a Guava^®^ easyCyte™ Flow Cytometer (Luminex Corporation, Austin, TX, USA).

### 2.11. CNDs Uptake 

THP-1 human monocyte-derived macrophages were grown to 85–90% confluence with corresponding media in clear cell plates and pre-treated with or without the following inhibitors for 30 min: Cytochalasin A or D (5 µg/mL), chlorpromazine (10 µg/mL), genistein (200 µM), nocodazole (20 µM), phenylglyoxal (100 µg/mL), amiloride hydrochloride (50 uM), n-phenylanthranilic acid (0.1mM), niflumic acid (10 mM), ebselen (15 µM), amiodarone hydrochloride (10 µM), chlorpromazine HCl (0.1 mg/mL), mercury chloride (0.075 mM), and copper sulfate (100 µM). Then, cells were treated with a concentration of 0.1 mg/mL CNDs for 24 h. Cells were harvested and resuspended in PBS. Fluorescence was read at 360/460 top 400 nm in a well plate reader (Synergy 2.0).

## 3. Results

### 3.1. Characterization of CNDs

**Figure 1:**
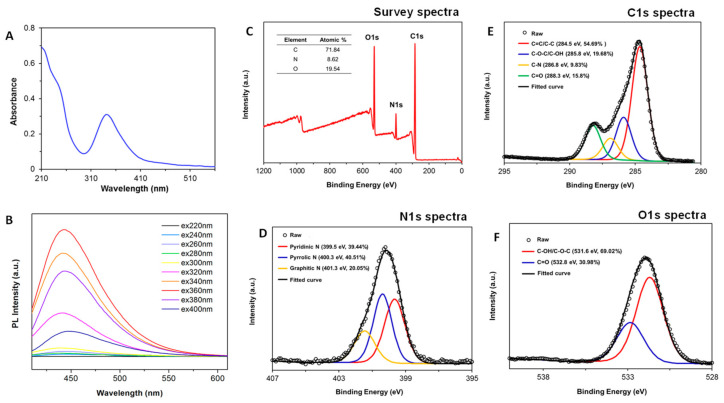
Characterization of CNDs. (**A**) Absorption spectrum. (**B**) CNDs show emission peak of ≈450 nm with excitation wavelengths from 220 to 400 nm. (**C**–**F**), X-ray photoelectron spectrum signals.

The UV-visible CNDs spectrum shows a shoulder peak at 240 nm, which is consistent with π–π∗ transitions of C–C and C = C bonds in sp^2^ hybrid regions. The main peak at 350 nm comes from the n–π∗ transitions of C=O moieties (Figure 1A). The fluorescence emission spectra at different excitation wavelengths starting from 220 to 400 nm with 20 nm intervals were conducted. The strongest emission peak is centered at 450 nm with an excitation wavelength of 350 nm. XPS was used to examine the surface functional groups of CNDs. The XPS survey spectra demonstrates characteristic peaks corresponding to C1s (284.5 eV), O1s (531.6 eV), and N 1s (399.5 eV), which confirms the presence of C, O, and N elements. Based on the high-resolution C1s XPS spectra, four components were detected at 284.5 eV (C=C/C-C, 54.69%), 285.8 eV (C-O-C/C-OH, 19.68%), 286.8 eV (C-N, 9.83%), and 288.3 eV (C=O, 15.8%). The high-resolution N1s XPS spectrum shows three peaks at 399.5, 400.3, and 401.3 eV, which can be attributed to pyridinic, pyrrolic, and graphitic nitrogen atoms. The results of deconvolution treatment for the high-resolution O1s spectrum of the sample shows two peaks, located at 531.6 eV and 532.8 eV, respectively, which were attributed to C–OH/C–O–C and C = O.

### 3.2. Differentiation of THP-1 Monocytes

**Figure 2:**
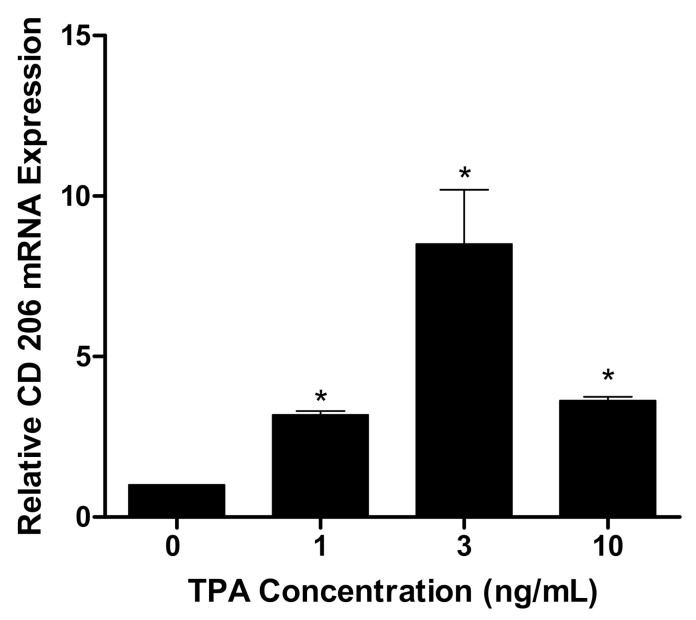
Increase in CD206 expression in THP-1 human monocyte-derived macrophages. THP-1 cells (3.3 × 10^6^) were treated with 0, 1, 3, and 10 ng/mL of 12-O-tetradecanoylphorbol 13-acetate (TPA) in RPMI media for 72 h, upon which media was refreshed. Then, cells were left to incubate and mature for another 72 h. RNA was isolated, converted to cDNA, and probed for CD206 using SYBR green qRT-PCR reagents via Biosystems™ StepOnePlus™ Software v2.3. GAPDH was the housekeeping gene. All data represent mean ± SEM. (*n* = 3, *, *p* < 0.05 vs. control).

THP-1 monocytes were treated with 0, 1, 3, and 10 ng/mL TPA for 72 h in RPMI media so as to stimulate the cells into differentiation. Then, the media was replaced, and cells were allowed to incubate for an additional 72 h. By way of qrt-PCR, expression of CD 206 (a macrophage differentiation marker) was assessed. As demonstrated by Figure 2, an increase in the expression of CD 206 (*p* < 0.05) was observed in these TPA-treated cells.

### 3.3. Cell Viability Determined by Trypan Blue Cell Counts and ViaCount Flow Cytometry

**Figure 3:**
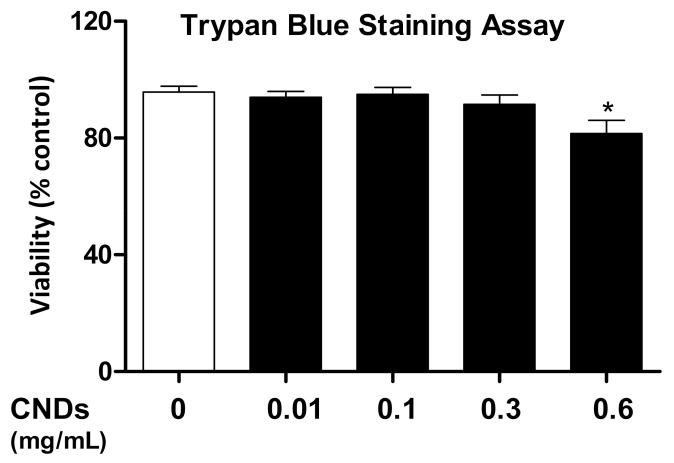
**Effect of CNDs on cell viability (Trypan Blue).** THP-1 cells were treated with 3 ng/mL TPA in the presence or absence of 0.01, 0.1, 0.3, or 0.6 mg/mL CNDs in RPMI media for 72 h, upon which media was refreshed. Then, cells were left to incubate for another 72 h. Cells were harvested, and a cell count performed using a hemocytometer and Trypan Blue. All data represent mean ± SEM (*n* = 3, *, *p* < 0.05 vs. control).

**Figure 4:**
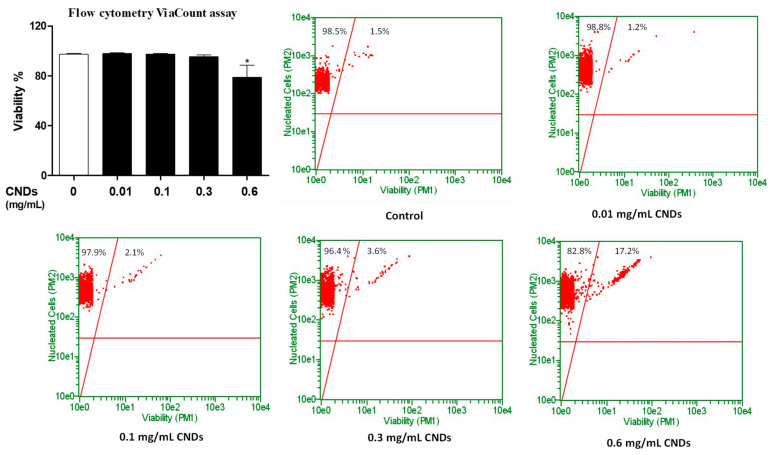
**Effect of CNDs on cell viability (ViaCount).** THP-1 cells were treated with 3 ng/mL TPA in the presence or absence of 0.01, 0.1, 0.3, or 0.6 mg/mL CNDs in RPMI media for 72 h, upon which media was refreshed. Then, cells were left to incubate for another 72 h, after which cells were harvested and treated with ViaCount reagent. Then, a viability analysis was performed using a Guava^®^ easyCyte™ Flow Cytometer (Single Sample System). All data represent mean ± SEM. (*n* = 3, *, *p* < 0.05 vs. control).

In order to analyze the effect of CNDs on the viability of THP-1 monocyte-derived macrophages, cell counts in a hemocytometer were performed using Trypan Blue and ViaCount Flow Cytometry. The general concept behind the cell counts is that Trypan Blue can enter cells with a compromised membrane [12]. ViaCount reagent demonstrates effects on cell viability by using two DNA-binding dyes. One stains DNA in all cells, the other specifically binds to DNA in dead cells. THP-1 cells were treated with CNDs concentrations ranging from 0.01 to 0.6 mg/mL for 72 h. After refreshing media, and an additional incubation period of 72 h, cells were analyzed with both methods. The Trypan Blue cell counts demonstrate a significant decrease (*p* < 0.05) in cell viability only at a concentration of 0.6 mg/mL (Figure 3). Flow cytometric analysis demonstrates a significant reduction (*p* < 0.05) in cell viability at 0.6 mg/mL CNDs (Figure 4), and also that the percentage of live cells in the upper left quadrant only differ significantly (*p* < 0.05) between untreated cells and cells treated with the same CNDs concentration (Figure 4).

### 3.4. Expression of M1/M2 Biomarkers in Macrophages as Affected by CNDs

M1 (pro-inflammatory) macrophages play a crucial intermediary role in the atherosclerosis disease state. The effect of CNDs on the expression of M1 or M2 biomarkers was analyzed by PCR. THP-1 monocytes were co-treated with 3 ng/mL TPA and 0.1 mg/mL CNDs for 72 h. Then, these cells had their media refreshed, followed by an additional incubation period of 72 h. Afterwards, cells were harvested, RNA isolated, cDNA synthesized, and analyzed for expression of genes by qrt-PCR.

**Figure 5:**
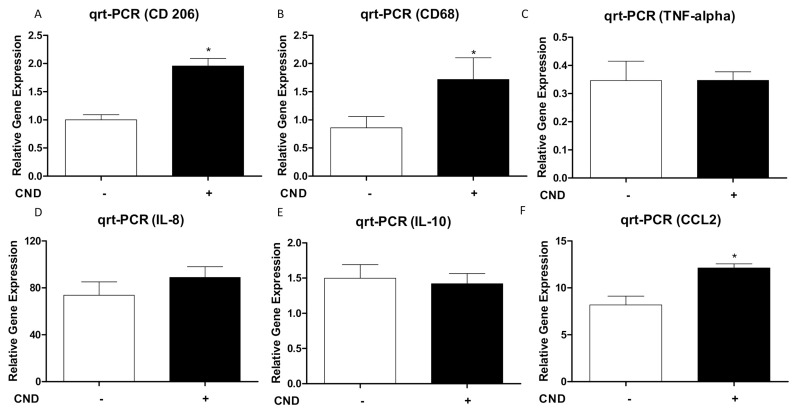
Effect of CNDs on expression of M1/M2 biomarkers in macrophages. THP-1 cells (1 × 10^6^) were treated with 3 ng/mL TPA in the presence or absence of 0.1 mg/mL CNDs in RPMI media for 72 h, upon which media was refreshed. Then, cells were left to incubate and mature for another 72 h. RNA was isolated, converted to cDNA, and probed for CD206 (**panel A**), CD68 (**panel B**), TNF-alpha (**panel C**), IL-8 (**panel D**), IL-10 (**panel E**), and CCL2 (**panel F**) using SYBR green qRT-PCR reagents via an Applied Biosystems™ StepOne™ Real-Time PCR System. GAPDH was the housekeeping gene. All data represent mean ± SEM. (*n* = 4–6, *, *p* < 0.05 vs. control).

As previously mentioned, CD206 is a recognized M2 biomarker. IL-10 cytokine, which suppresses the immune response, was also analyzed as an M2 biomarker [13]. A selection of M1 biomarkers was included in the analysis. IL-8 and TNF-α are all well-established pro-inflammatory cytokines, and they are also regarded as M1 biomarkers. CCL2 serves as a macrophage chemoattractant [14,15]. CD68 is a surface receptor classified as an M1 biomarker. Our results indicate a significant increase (*p* < 0.05) in CD 206, CD 68, and CCL2 expression in cells treated with 0.1 mg/mL CNDs. No significant effect was observed in the expression of TNF-alpha, IL-8, and IL-10 (Figure 5).

### 3.5. CNDs Effect on the Phagocytic Activity of Macrophages

**Figure 6:**
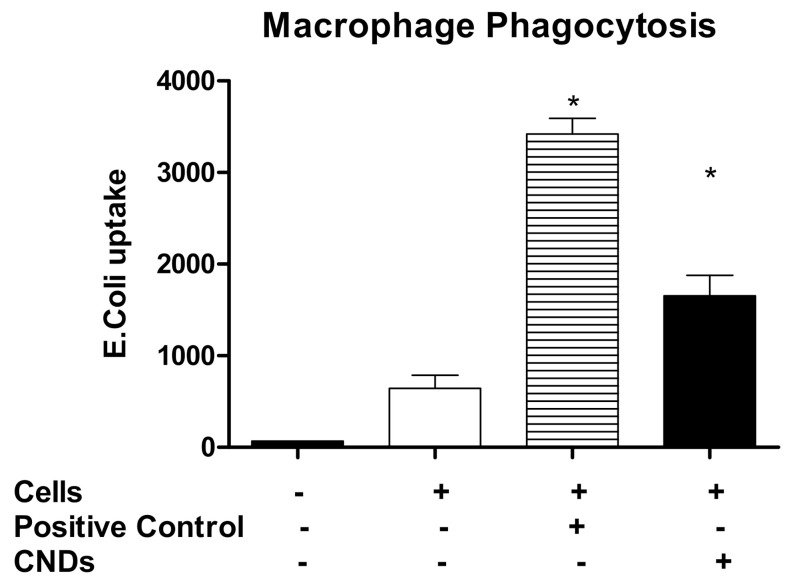
Increase in phagocytic activity in CND co-treated cells. THP-1 cells (2 × 10^6^) were treated with 3 ng/mL TPA in the presence or absence of 0.1 mg/mL CNDs. Cells were incubated for a period of 72 h, upon which media was refreshed, and followed by another incubation period of 72 h. Cells were harvested, and a sample of control cells were treated with 1000 ng/mL TPA to serve as a positive control. Cells were distributed in a 96-well plate and left to incubate for 18 h. Treatment of cells with fluorescent *E. coli* suspension followed for 2 h, upon which the suspension was removed. Cells were finally treated with Trypan Blue. The removal of Trypan Blue preceded the reading in a BioTek™ Synergy 2.0 plate reader. All data represent mean ± SEM. (*n* = 5, *, *p* < 0.05 vs. control).

Phagocytic activity is an essential function of macrophages. Macrophages that absorb an excess of ox-LDL turn into foam cells, which are the main component of the necrotic plaque that settles on an artery bed [16]. In order to analyze the effect of CNDs on the phagocytic function of THP-1 monocyte-derived macrophages, THP-1 cells were co-treated with 3 ng/mL TPA and with or without CNDs at 0.1 mg/mL with similar incubation periods as denoted previously. Cells were harvested and incubated in a 96-well plate for 18 h. Before this incubation period, a sample of control cells was treated with 1000 ng/mL TPA so as to activate macrophages (positive control). Next, cells were treated for 2 h with a suspension of fluorescent-labeled *Escherichia coli*. Lastly, cells were treated with a Trypan Blue suspension for 1 min before analysis in a plate reader. Our results indicate that THP-1 monocytes treated with 0.1 mg/mL CNDs during the differentiation process exhibit a significant increase (*p* < 0.05) in phagocytic activity (Figure 6).

### 3.6. Potential Uptake Routes of CNDs into Macrophages

In order to exert intracellular effects, xenobiotics often need to cross the plasma membrane. Nanoparticles are an example of xenobiotics, and recently, uptake routes have become characterized. Hara et al. demonstrated that pre-treatment of THP-1 monocyte-derived macrophages with cytochalasin D, a potent inhibitor of actin polymerization, led to a decrease in the uptake of nano-silica particles [17]. This supports the notion that nanoparticles may mainly enter the cell through phagocytosis.

**Figure 7:**
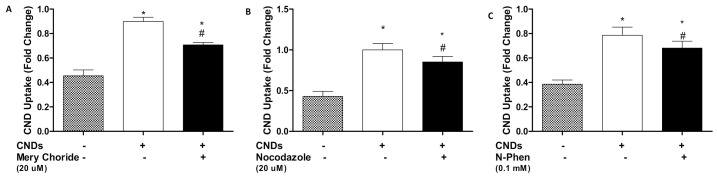
Effect of mercury chloride (**A**), nocodazole (**B**) and N-phen (**C**) on the uptake of CNDs into macrophages. THP-1 human monocyte-derived macrophages were treated for 30 min with or without inhibitors (for 15 min). Next, cells were treated with 0.1 mg/mL CNDs for 24 h. Lastly, cells were harvested and placed in a 96-well plate. Fluorescence analysis ensued in a BioTek™ Synergy 2.0 plate reader. All data represent mean ± SEM. (*n* = 4, *, *p* < 0.05 vs. control, #, *p* < 0.05 vs. CND treatment only).

**Figure 8:**
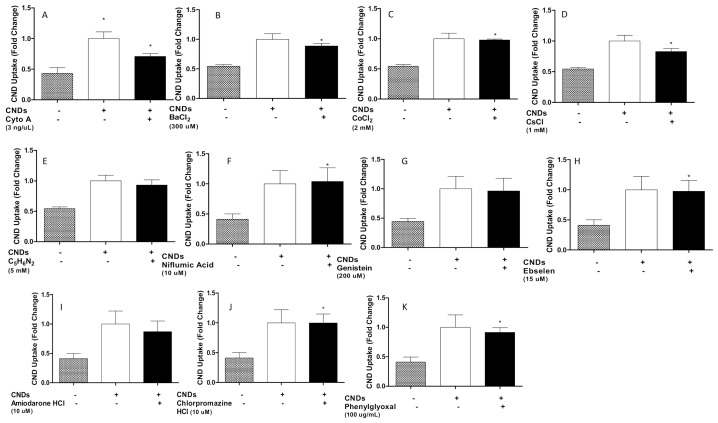
Chemical inhibitors’ effect on uptake of CNDs into macrophages (**Panel A**: cyto A; **Panel B**: BaCl_2_; **Panel C**: CoCl_2_; **Panel D**: CsCl; **Panel E**: C_5_H_6_N_2_; **Panel F**: Niflumic Acid; **Panel G**: Genistein; **Panel H**: Ebselen; **Panel I**: Amiodarone HCl; **Panel J**: Chlorpromazine HCL; **Panel K**: Phenylglyoxal). THP-1 monocyte-derived macrophages (1 × 10^6^) were treated for 30 min with or without inhibitors. Next, cells were treated with 0.1 mg/mL CNDs for 24 h. Lastly, cells were harvested and placed in a 96-well plate. Fluorescence analysis ensued in a BioTek™ Synergy 2.0 plate reader. All data represent mean ± SEM. (*n* = 3, *, *p* < 0.005 vs control).

In order to characterize potential uptake routes of CNDs into macrophages, THP-1 monocytes with 3 ng/µL TPA with incubation periods were differentiated as described previously. Treatment with or without a variety of chemical inhibitors (Table 2) for 30 min ensued (with the exception of mercury chloride for 15 min) before treating cells with 0.1 mg/mL CNDs. Then, cells were harvested, placed in a 96-well plate, and analyzed for fluorescence in a plate reader. Our results indicate significant inhibition (*p* < 0.05) of CNDs uptake with the use of Nocodazole, mercury chloride, and N-phenylanthranilic acid (Figure 7a–c). All other inhibitors used did not show a significant inhibition in CNDs uptake (Figure 8a–k).

**Figure 9:**
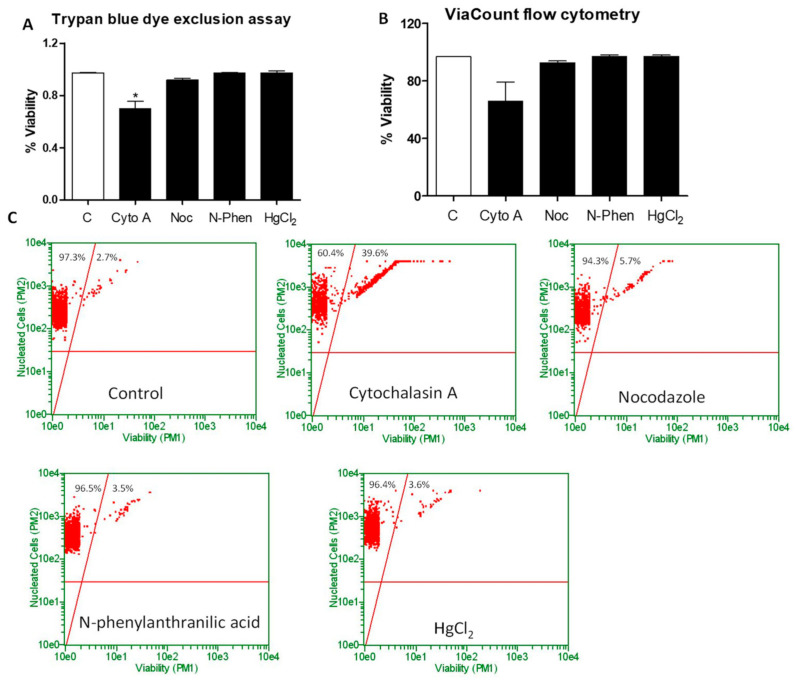
Effect of chemical inhibitors on the viability of cells (ViaCount). THP-1 monocyte-derived macrophages (1 × 10^6^) cultured as mentioned previously, then harvested, resuspended in HBSS, and treated for 30 min with cytochalasin A (3 µg/mL), nocodazole (20 mM), N-phenylanthranilic acid (0.1 mM), or mercury chloride (0.075 mM) for 15 min. With media refreshed. **Panel A**: Then, cells were incubated for a period of 24 h, after which a viability analysis was performed with a hemocytometer cell count using Trypan Blue. **Panel B**,**C**, cells were incubated for a period of 24 h before treatment with ViaCount reagent. Then, a viability analysis was performed using a Guava^®^ easyCyte™ Flow Cytometer (Single Sample System). All data represent mean ± SEM. (*n* = 3, *, *p* < 0.05 vs. control).

With data indicating inhibition in CNDs uptake of cells treated with nocodazole, n-phenylanthranilic acid, and mercury chloride, the effects of these inhibitors on cell viability were determined using flow cytometry. Cells were differentiated as previously described. Treatment with the previously mentioned inhibitor concentrations followed. After refreshing culture media (so as to remove the presence of the inhibitors), cells were left to incubate for 24 h. Cell viability was tested using previously mentioned Trypan Blue cell count and ViaCount protocols. Our results indicate no significant decrease in the viability of macrophages at any designated concentration of each inhibitor in both the Trypan Blue (Figure 9a) and ViaCount analyses (Figure 9b,c). Representative flow cytometric analysis demonstrates no change in the percentage of live cells present in the upper left quadrant for any cells treated with inhibitors (Figure 9c).

## 4. Discussion

Macrophages play an important role as mediators of atherosclerosis. For this reason, they are highly sought targets when studying the disease state. CNDs are recently discovered carbon-based nanomaterials reported to have sizes of 10 nm or less, and they also exhibit favorable qualities for use in biomedical application [27]. Collectively, our study represents an initiatory attempt at understanding the interactions of CNDs and macrophages involved in atherosclerosis. Our analysis included studying changes in macrophage biomarker expression. In addition, we studied the effect of CNDs on the phagocytic activity of macrophages. Lastly, we investigated possible uptake routes of this nanoparticle.

Macrophages play a crucial intermediary role in the atherosclerosis disease state. The overabundance of settling macrophages and foam cells, due to an excess of lipoprotein ingestion, leads to the emergence of plaque. These macrophages exacerbate the inflammatory microenvironment by secreting pro-inflammatory cytokines to different cell types [28]. A side effect of this process is an excessive dysregulation of macrophage polarization, causing circulating monocytes to differentiate into pro-inflammatory macrophages (M1) in abundance.

As a model, THP-1 human monocyte-derived macrophages were utilized. These monocytes exhibit a homogenous genetic background and differentiate into adheren macrophages upon exposure to 12-O-tetradecanoylphorbol-13-acetate (TPA). The cell line is resembling of primary monocytes/macrophages, which made it an ideal model for our purposes [29]. These macrophages are characterized by an increase in the expression of scavenger receptors while simultaneously reducing LDL receptor expression [30]. Due to their ability to absorb modified lipoproteins and convert to foam cells, THP-1 monocyte-derived macrophages act as a representative model to study macrophage involvement in atherogenesis. In fact, this model has seen extensive use in recent years, appearing in several in vitro studies regarding monocyte/macrophage drug transport, signaling, and function [31]. The favorable increase in CD 206 (a macrophage biomarker) expression observed at 3 ng/µL TPA confirmed monocyte differentiation (Figure 2). With this result, and previously mentioned properties, THP-1 monocyte-derived macrophages became a useful model to analyze the effects of CNDs on the phagocytic activity of macrophages and their expression of biomarkers.

Phagocytic activity is among the most important functions of macrophages. As a form of endocytosis, phagocytosis is defined by the use of a cell membrane to engulf extracellular particles, allowing them entrance into the cell’s cytoplasm. As key players of the immune system, macrophages ingest a variety of particles including microbes, modified lipids, and even dead cells entirely [32]. The phagocytic function of macrophages, as well as other roles in immunological responses, makes macrophages a popular target for therapeutic testing. Despite this popularity, no research has been committed to studying the effects of CNDs on the phagocytic activity of primary macrophages. Our study provides a novel insight into this matter. As shown in Figure 6, THP-1 monocyte-derived macrophages that were treated with 0.1 mg/mL CNDs during the differentiation process exhibit an increase in phagocytic activity.

The expressions of macrophage biomarkers, from treatment with CNDs during the differentiation process of THP-1 monocyte-derived macrophages, were analyzed to further understand if this boost in phagocytic function favors M1 or M2 polarization. Circulating monocytes that are activated through receptor-ligand binding differentiate into M1 (pro-inflammatory) or M2 (anti-inflammatory) macrophages. This is typically dependent on the immunological response in need. M1 macrophages typically eliminate xenobiotics through phagocytosis and promote the local inflammatory environment. In an atherogenic state, M1 macrophages aim to phagocytose modified lipoproteins in an effort to clear cholesterol. They also extend the inflammatory response by secreting several pro-inflammatory cytokines such as TNF-alpha, IL-1, IL-6, and IL-12 [33]. These cytokines signal additional circulating monocytes to differentiate into M1 macrophages, as well as a host of other cell types involved in immunity. In addition to the previously mentioned cytokines, M1 macrophages exhibit a variety of biomarkers. In vitro studies identify M1 macrophages by the up-regulation of certain receptors such as CD 64, 68, and 80 [34,35]. Though crucial for host defense, the functions of M1 macrophages can be expropriated during disease states, resulting in dysregulated inflammation [36]. In contrast, M2 macrophages promote tissue repair, clear cellular debris, and secrete anti-inflammatory cytokines [37]. The presence of M2 macrophages is associated with regressing plaques. Biomarkers of M2 macrophages include anti-inflammatory cytokines such as IL-4, IL-10, and IL-13, as well as a variety of surface receptors that include CD 206, CD 23, and CD 163. As a commonality, both types exhibit phagocytic function.

Cells treated with 0.1 mg/mL CNDs demonstrated a significant increase in CCL-2 and CD 68, which are both considered M1 biomarkers (Figure 5b,f). Several studies have demonstrated that M1 macrophages accumulate cholesterol via modified, atherogenic LDL (e.g., ox-LDL) as opposed to native LDL [28]. Modified LDL is internalized through phagocytosis. In the case of atherosclerosis, M1 macrophages recognize ox-LDL by means of scavenger receptors including scavenger receptor A, CD 36, and CXCL16 [28,38]. CD 68 and its mouse ortholog macrosialin have also been recognized as receptors for ox-LDL [39]. The excessive uptake of cholesterol from modified lipoproteins leads to a dysregulation of lipid metabolism within M1 macrophages. This dysregulation results in a build-up of free cholesterol, which is toxic unlike other forms such as cholesteryl ester [38]. Among the effects of free cholesterol is the activation of stress responses in the endoplasmic reticulum, which prevents the re-esterification of cholesterol. Normally, macrophages submit cholesterol through a process of esterification that permits a series of transporters to expel them from the cell [28]. Thus, ER stress promotes the build-up of free cholesterol in macrophages, which in turn furthers the creation of foam cells. This knowledge, combined with the increase in both M1 biomarkers observed, would seem to suggest CNDs tilt the polarization of macrophages toward M1.

Our results also demonstrated a significant increase in the expression of CD 206, which is a prominent M2 biomarker (Figure 5a). This receptor has functionality in the phagocytosis of different bacteria [13]. The increase observed in expression of this receptor may very well explain the increase observed in phagocytic activity, considering that CD 206 recognizes *E. coli* [40]. Additionally, CD 206 serves as a regulator of adipocyte progenitors [41]. This result seemingly counters the increase in M1 biomarkers mentioned previously and suggests polarization toward M2 phenotypes.

In recent years, interest in the development of various carbon nanoparticles in the biological application has risen. For example, diamond-like carbon (DLC) nanofilm is a promising material for application in medical implants, with high mechanical and chemical inertness and biocompatibility [42,43]. Both cell culture and animal experiments have shown that DLC coating does not cause toxicity and inflammation [44]. The colloidal solution of nanocarbon has recently been shown to inhibit bacteria’s growth without affecting the viability of eukaryotic animal cells [44]. Studies by Jelinek et al. have shown that Ge-doped DLC layers with low doping levels are not cytotoxic, while for higher doping levels, Ge has been proven to be cytotoxic, which is related to the production of reactive oxygen species [42,43]. Carbon nanotubes (CNTs) are promising candidates in nanomedicine in treating various diseases [45]. However, these nanotubes have been shown to exert various toxic effects on various cells. Multi-walled carbon nanotubes (MWCNT) cause dose-dependent cytotoxicity when its concentration in THP-1 monocyte-derived macrophages is higher than 25 µg/mL, and the concentration in lung epithelial cell-derived A549 cells is higher than 100 µg/mL [46]. In our studies, trypan blue cell count and ViaCount flow cytometry assays showed that CNDs have no effect on cell viability of THP-1 monocyte-derived macrophages at concentrations that do not exceed 0.3 mg/mL (300 µg/mL). These results suggested that CNDs showed relatively low toxicity and better biocompatibility compared to carbon nanotubes.

To further deepen our understanding of the interaction of CNDs and macrophages, the final aims of this study examined potential uptake routes of CNDs into THP-1 monocyte-derived macrophages. Previous studies have denoted the involvement of actin, microtubules, and endocytic pathways in the uptake of nanoparticles: (i) Dos Santos et al. showed that the use of chlorpromazine, genistein, nocodazole, and cytochalasin A inhibited the uptake of carboxylated polystyrene nanoparticles via clathrin-mediated endocytosis in various cell lines [21,47]. Chlorpromazine suppresses clathrin disassembly and receptor recycling in the cell membrane. Genistein specifically inhibits tyrosine kinase receptors involved in calveolae-mediated endocytosis. Nocodazole and cytochalasin A disrupt microtubule and actin filaments. (ii) Park et al. demonstrated that amiloride successfully inhibited the uptake of hydrophobically modified glycol chitosan nanoparticles (HGC-NPs). Amiloride inhibits macropinocytosis by suppressing Na^+^/H^+^ exchange [20]. These studies suggest that nanoparticles may enter cells primarily through endocytic pathways. Nonetheless, no research has been committed to utilizing these inhibitors to characterize the potential uptake routes of CNDs into macrophages.

In addition to the previously mentioned inhibitors, our study employed a variety of chemical inhibitors designed to cover multiple cellular entrance routes. Among the list were mercury chloride (HgCl_2_), which is known to inhibit aquaporin channels [21]. Barium chloride and 4-aminopyridine also served to block potassium channels [18]. The extensive list of inhibitors also included niflumic acid, ebselen, and phenylglyoxal. Uptake analysis demonstrated significant inhibition in the uptake of CNDs when macrophages were treated with nocodazole, N-phenylanthranilic acid, and mercury chloride (HgCl_2_) (Figure 7). Changes were also observed with other inhibitors; however, no significant trend could be established (Figure 8). Treatment with cytochalasin A demonstrated inhibition of CNDs uptake. However, upon performing cell viability tests, it was discovered that cytochalasin A had adverse effects on macrophages (Figure 9). This likely represents the observed inhibition of CNDs uptake as a causation of cell death, which would reduce the fluorescent signal of CNDs, giving the appearance of uptake inhibition.

The observed inhibition of CNDs uptake as a result of treatment with nocodazole suggests that CNDs can gain entrance into cells through endocytic pathways. N-phenylanthranilic acid acts as a chloride channel blocker in cell membranes. The CNDs utilized in this study exhibit negatively charged surface functional groups. Given that chloride is a negatively charged molecule, the passage of CNDs through this channel has merit. This result also gives rise to an interesting notion. Though small even in the nanoparticle scale (≈10 nm), CNDs are still relatively large in comparison to chloride ions (≈0.2 nm). Our results suggest that depending on the surface groups tailored to CNDs, size may not be an issue in gaining entrance into cells through ion channels. Nanoparticles have demonstrated the capability of binding to carrier proteins in order to enter plant cells through aquaporins, ion channels, or endocytosis [48]. These findings explain the inhibition of uptake observed with treatment of macrophages with HgCl_2_ and suggest that CNDs may pass through aquaporins in similar fashion. 

Our results showed that CNDs are taken up by THP-1 monocyte-derived macrophages. However, in vivo, it remains unclear whether CNDs can enter macrophages and accumulate in the aorta or atherosclerotic plaque. In addition, the efficacy of the interaction of nanoparticles and their target cell is judged not just by their ability to enter a cell but also by the time it takes to be metabolized or released. It is not yet clear whether CNDs are released from macrophages or other cells or tissues and whether this nanoparticle can be metabolized into different molecules, which remains to be further examined in the future.

In summary, our results provide novel evidence of the interaction of CNDs and macrophages involved in atherosclerosis. CNDs were confirmed to be non-toxic in concentrations that do not exceed 0.3 mg/mL by performing Trypan Blue cell counts and ViaCount flow cytometry. Our PCR results indicate a significant increase in the expression of at least one M2 biomarker (CD 206) and increases in M1 biomarkers CCL2 and CD 68. Two of these biomarkers are involved in the phagocytic function of macrophages. Although no fixed conclusions can yet be assumed regarding how CNDs affect macrophage polarization, our phagocytosis assay results indicate that CNDs treatment during the differentiation process boosts phagocytic activity, which is possibly due to the scavenging of ROS. Lastly, we also determined potential cellular uptake routes of CNDs. Results showcased inhibitions of CNDs uptake in cells treated with nocodazole, n-phenylanthranilic acid, and mercury chloride, providing evidence for entrance routes in the form of endocytosis, chloride, and water channels. Due to their crucial role as mediators in atherogenesis and their involvement in several aspects of the immune response, macrophages are popular targets in vascular research and therapeutic treatment [49]. Upon injury to the endothelium of blood vessels by ox-LDL, circulating monocytes differentiate into pro-inflammatory (M1) or anti-inflammatory (M2) macrophages [49]. In progressing lesions, M1 macrophages engulf excess ox-LDL [49]. The effect of CNDs on the polarization of macrophages has not been examined. Our results would provide new information on the potential applications of novel CNDs to modulate macrophages’ polarization, which is a promising treatment strategy for atherosclerosis, a chronic progressive inflammatory disease. Collectively, these results yield a deeper understanding in the interaction between macrophages involved in atherosclerosis and CNDs.

## Figures and Tables

**Table 1:** Primer sequences for qrt-PCR reactions.

Target	Forward Primer	Reverse Primer
GAPDH	5’-AGA ACG GGA AGC TTG TCA TC-3’	5’-GGA GGC ATT GCT GAT GAT CT-3’
IL-8	5’-CTC TGT GTG AAG GTG CAG TT-3’	5’ –AAA CTT CTC CAC AAC CCT CTG-3’
CCL-2	5’-GCT CAG CCA GAT GCA ATC AA-3’	5-GGT TGT GGA GTG AGT GGT CAA G-3’
CD68	5′-TCAGCTTTGGATTCATGCAG-3′	5′-AGGTGGACAGCTGGTGAAAG-3′
IL-10	5′-CTAACCTCATTCCCCAACCA-3′	5′-GTAGAGACGGGGTTTCACCA-3′
TNF-α	5′-CTATCTGGGAGGGGTCTTCC-3′	5′-GGTTGAGGGTGTCTGAAGGA-3′

**Table 2:** Inhibitors used for potential uptake routes of CNDs.

Inhibitor Name	Abbrev	Function
4-Aminopyridine ~98%	C_5_H_6_N_2_	Ion channel blocker (K^+^) [18]
Amiodarone Hydrochloride	Amiodarone HCL	Non-selective ion channel blocker [19]
Barium Chloride Anhydrous	BaCL_2_	Ion channel blocker (K^+^) [18]
Chlorpromazine HCL	Chlorpromazine HCL	Suppresses clathrin disassembly [20,21]
Cobalt (II) Chloride	CoCL_2_	Ion channel blocker (Ca^+^) [22]
Copper Sulfate	Cu	hAQP3 Aquaporins [23]
Cytochalasin A	Cyt	Actin disruptor [21]
Ebselen	Ebselen	Inhibits mammalian H^+^, K^+^–ATPase [24]
Genstein	Genstein	Inhibits tyrosine kinase receptors [21]
Mercury Chloride	Mercury Chloride	hAQPI Aquaporins [23]
N-Phenlanthranilic Acid	N-Phen	Ion channel blocker (Cl^−^) [25]
Niflumic Acid	Niflumic Acid	Ion channel blocker (Cl^−^)
Nocodazole	Phenylglyoxal	Actin and microtubule disruptor [21]
Phenylglyoxal	Phenylglyoxal	Selective inhibitor of phagocytosis [26]

## Data Availability

The data used to support the findings of this study are available from the corresponding author upon request.
